# Nurse compliance with personal protective equipment when handling chemotherapy: a multicenter cross-sectional study in Palestine

**DOI:** 10.1186/s12913-026-14132-x

**Published:** 2026-02-03

**Authors:** Nader Lama, Hanan Saca-Hazboun

**Affiliations:** 1 Faculty of Nursing and Health Sciences, Bethlehem University, 5 Rue des Frères, Bethlehem, Palestine; 2 Oncology and Palliative Care Consultant, Faculty of Nursing and Health Sciences, Bethlehem University, 5 Rue des Frères, Bethlehem, Palestine

**Keywords:** Chemotherapy, Oncology nurses, Personal protective equipment, Compliance

## Abstract

**Background:**

Occupational exposure to chemotherapy poses acute and long-term health risks, including cancers. Personal protective equipment is fundamental to reducing these risks; however, compliance among nurses is still inadequate worldwide. Most existing studies have occurred in industrialized countries, and no published studies have examined compliance with personal protective equipment by oncology nurses in Palestine. This study aimed to assess compliance with personal protective equipment and identify factors influencing compliance.

**Methods:**

A cross-sectional descriptive design was employed. One hundred oncology nurses (68.5% response rate) from six Palestinian hospitals completed the Hazardous Drug Handling Questionnaire. A Pearson correlation analysis explored relationships between compliance with personal protective equipment and independent variables.

**Results:**

Nurses demonstrated moderate compliance with personal protective equipment (mean = 2.3, standard deviation [SD] = 0.67), with the highest compliance during chemotherapy administration (mean = 2.41, SD = 0.68) and the lowest during disposal (mean = 2.2, SD = 0.90). Influencing factors included insufficient knowledge of chemotherapy exposure risks, perceived barriers to using personal protective equipment, low self-efficacy, moderate perceived exposure risk, and a moderate workplace safety climate. Compliance improved among those with oncology certification (*p* = 0.005) and those who received on-the-job training (*p* = 0.03).

**Conclusions:**

Moderate compliance with personal protective equipment indicates that oncology nurses remain at risk of exposure to chemotherapy. Targeted interventions are required, including continuous education, structured training in safe handling, and developing a supportive workplace culture through monitoring and reinforcement of safety measures. These findings present evidence to guide policymakers and healthcare leaders in strengthening chemotherapy safety standards in Palestinian healthcare centers.

**Supplementary Information:**

The online version contains supplementary material available at 10.1186/s12913-026-14132-x.

## Background

Chemotherapy is commonly used to cure cancer and contributes to enhanced clinical outcomes, including improved quality of life and prolonged survival [1–3]. However, several chemotherapeutic agents are themselves carcinogenic or potentially carcinogenic according to the International Agency for Research on Cancer [4].

With the growing number of cancer diagnoses, the likelihood of environmental exposure among health workers is rising [1, 2]. Nurses in oncology settings frequently handle chemotherapy, placing them at particular risk [5]. This study concentrates on nurses because of their prolonged, repeated interactions with patients receiving treatment.

Occupational exposure can occur during the preparation, administration, and disposal of chemotherapy drugs and the handling of contaminated body waste [5, 6]. Surface contamination and needlestick injuries add to exposure incidents [7]. Before the introduction of safe-handling guidelines by the American Society of Health-System Pharmacists in 1983 and the Occupational Safety and Health Administration (OSHA) in 1986, direct exposure was associated with multiple acute and long-term effects [5]. Acute effects may manifest as skin rashes, allergic reactions, alopecia, nausea, vomiting, and cardiotoxicities [1, 2, 8], while chronic outcomes include infertility, fetal harm [2], miscarriage, congenital abnormalities, and increased cancer risk [1].

Although harmful effects were recognized in the 1970s [1], evidence of elevated levels of mutagenic substances among nurses handling chemotherapy emerged later [2, 9]. Certain chemotherapy drugs, including alkylating agents, have been associated with secondary leukemia in treated patients [10, 11]. While treatment benefits outweigh the risks for patients, minimizing occupational exposure within the healthcare sector is fundamental.

Ongoing concerns for nurse safety have led multiple institutions, including OSHA, to publish safe-handling instructions recommending a hierarchy of controls to minimize hazards [12]. Personal protective equipment (PPE) represents the final layer of defense and provides a barrier between hazardous drugs and personnel [8]. Examples include goggles, masks, gowns, and gloves, all of which can effectively reduce exposure when used properly [2]. Professional organizations, such as the Oncology Nursing Society, recommend special chemotherapy gloves and gowns, double gloving, respirator masks when inhalation exposure is possible, and eye protection when splashing is a risk [13].

Despite clear guidelines and evidence supporting PPE’s effectiveness [14–17], compliance remains suboptimal globally [5, 8]. Studies report low use of gowns and goggles during administration and disposal activities [6, 15, 18], with the lowest use observed during the handling of body fluids [17]. A positive safety culture and lower nurse-to-patient ratios can improve compliance [19]. Reasons for low compliance are not fully understood [12, 20] but may relate to the voluntary nature of guidelines and reliance on institutional policies.

Most PPE compliance studies have been carried out in industrialized countries [2, 18], and little is known about compliance in low-resource settings such as Palestine. Oncology services in Palestine are mainly provided in governmental hospitals, characterized by fragmented treatment modalities and poor access to radiotherapy [21]. In the West Bank specifically, oncology units lack specialized pathology laboratories and advanced diagnostic technology. Within these units, chemotherapy is prepared by pharmacists, while nurses are responsible for administration in inpatient wards as well as outpatient clinics.

**Fig. 1:**
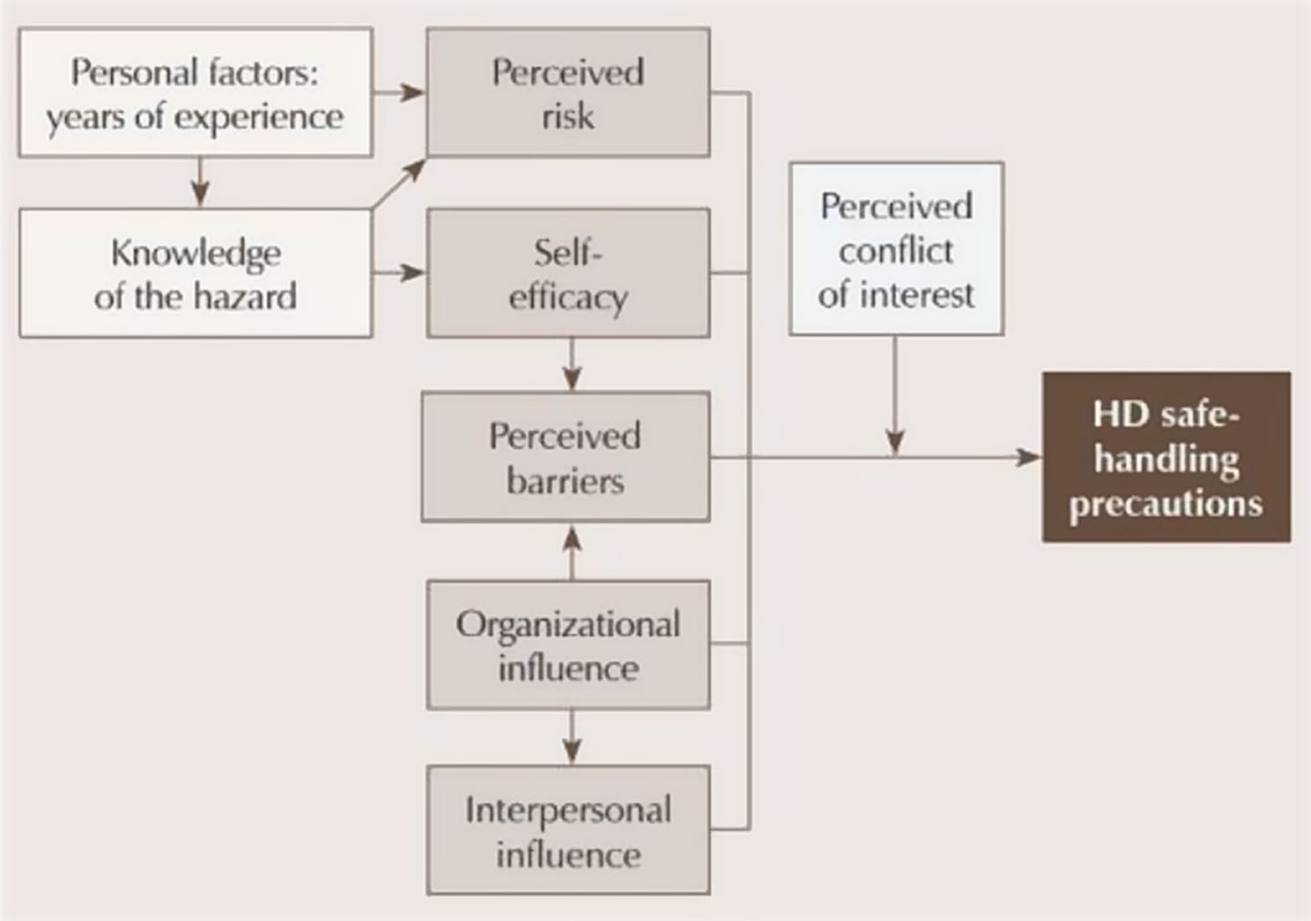
Factors predicting use of Hazardous Drug Safe-handling Precautions model. Legends: adapted model of factors influencing PPE compliance during chemotherapy handling, based on the original model of safe-handling precaution use. HD = hazardous drugs. The model was adapted with permission

This study was guided by the Factors Predicting Use of Hazardous Drug Safe-Handling Precautions theoretical framework [17]. The framework (Fig. 1) proposes that knowledge of the hazard, in this case chemotherapy, is associated with self-efficacy in using PPE and perceived exposure risk. Greater self-efficacy, as well as positive organizational influences, reduce perceived barriers. Perceived risk, self-efficacy, perceived barriers, organizational influences, interpersonal influences, and conflict of interest between providing patient care and ensuring self-protection are all factors that determine PPE compliance [5, 17].

To date, no published studies have evaluated PPE compliance among oncology nurses in Palestine or explored the factors influencing it. This study assessed PPE compliance and examined factors associated with it among oncology nurses in Palestine. In this study, compliance was defined as wearing protective gloves, disposable gowns, face masks, and eye shields in accordance with universal safety guidelines.

## Methods

### Design

This study employed a descriptive, cross-sectional quantitative design.

### Setting

The study was carried out in six hospitals in the West Bank and East Jerusalem where chemotherapy services are available, ensuring comprehensive coverage of nurses handling chemotherapy. These included two non-profit, one private, and three governmental hospitals, distributed across the southern, central, and northern governorates. The researchers did not have access to the Gaza Strip. Adult and pediatric hematology and oncology inpatient units, as well as outpatient departments, were included. Hospital names were not disclosed at the request of administrators.

### Study population

The target population included registered nurses in the West Bank and East Jerusalem who handle chemotherapy in the six hospitals providing such services (*N* = 172). Each hospital’s Nursing Administration Department confirmed the number of nurses (see Additional File 4).

### Inclusion and exclusion criteria

Eligible participants were oncology registered nurses responsible for chemotherapy administration and disposal, with at least one year of chemotherapy experience. Nurses with under one year of experience were excluded, as some hospitals require completion of internal chemotherapy training before administration. Practical nurses were excluded because chemotherapy handling is outside their scope of practice.

### Sampling technique and sample size

Total population sampling was used to recruit all nurses who handled chemotherapy in the participating hospitals and met the inclusion criteria. Of the 172 nurses, 146 were eligible, and questionnaires were distributed to all of them. A total of 100 completed questionnaires were returned, forming the final sample. Total population sampling was selected because the target population was small and well-defined, allowing all eligible individuals to be included. Therefore, a power analysis was not required.

### Data collection

Data were collected over three weeks (February 19-March 12, 2023). Formal emails were sent to hospital administrators, followed by visits to Nursing Administration Departments to explain study procedures.

Gatekeepers (Head Nurses) were assigned in each hospital to assist with the distribution and collection of questionnaires due to geographical distance and to minimize coercion. Eligible nurses were approached in person, provided with verbal and written study information, and invited to participate. Nurses who agreed signed informed consent and received the questionnaire with a return envelope. Sealed collection boxes were placed in each department for anonymous return. Questionnaires were retrieved at the conclusion of the data collection period.

### Instrument

Data were collected using Polovich and Clark’s Hazardous Drugs Handling Questionnaire [17] (see Additional File 1). Both the questionnaire and its associated theoretical framework were used with permission (see Additional File 2) and included demographic items as well as scales assessing constructs within the theoretical model. Minor wording modifications were made to suit the local context.

The questionnaire was distributed in English because a validated Arabic version was not available at the time of data collection. This was deemed appropriate since higher education and professional nursing training in Palestine are conducted in English. A pilot test with 20 nurses confirmed clarity and suitability; these participants were excluded from the final sample.

The content validity in the original development study was checked using the Content Validity Index (CVI). Three specialists in handling chemotherapy rated each item’s relevance on a scale of 1 to 4, with 1 indicating not relevant, 2 indicating somewhat relevant, 3 indicating fairly relevant, and 4 indicating very relevant.

Ratings of 1 or 2 were classified as “not relevant,” while ratings of 3 or 4 were “relevant.” The CVI was calculated for each item and for the overall scale using the universal agreement technique. Several items were revised following the initial assessment because of their low item-level CVI. A second assessment was conducted, after which all items achieved a CVI of 1.0 [17]. Additionally, Cronbach’s alpha values ranged from 0.72 to 0.95 [17]. The scoring system and interpretation guidelines were provided directly by Dr. Martha Polovich.

### Study variables

Independent variables included the seven constructs outlined in the theoretical model: knowledge of chemotherapy exposure, self-efficacy for using PPE, perceived exposure risk to chemotherapy drugs, perceived barriers to using PPE, workplace safety culture, interpersonal influences, and conflicts of interest. The dependent variable was PPE compliance. Table 1 presents operational definitions of the study variables.

**Table 1:** Operational definitions of study variables

Variable	Instrument	# Items/Scoring	Interpretation
*Outcome Measure*			
Compliance with PPE	HDHQ	Administration: 6 items Disposal: 5 items Handling Excreta: 5 items and Total precautions 0 = never to 5 = always Range 0–5 (Mean)	Higher score indicates higher compliance
*Independent Variables*			
Knowledge of the Hazard	Chemotherapy Exposure Knowledge	12 items: True, false, don’t know. Items 3, 6, 8, 9, 11 are false; all others are true. Correct answers = 1, all others = 0 Range: 0–12 (Sum of items)	Higher score indicates higher knowledge
Self-Efficacy	Self-efficacy for Using PPE	7 items, 4 = strongly disagree to 1 = strongly agree Items are reverse scored Range: 7–28 (Sum of items)	Higher score indicates higher self-efficacy
Perceived Barriers	Barriers to Using PPE	13 items, 1 = strongly disagree to 4 = strongly agree Range: 13–52 (Sum of items)	Higher score indicates higher perceived barriers
Perceived Risk	Risks of Chemotherapy Exposure	7 items, 1 = strongly disagree to 4 = strongly agree Range: 7–28 (Sum of Items)	Higher score indicates higher perceived risk
Organizational Influences	Workplace Safety Climate	21 items, 1 = strongly disagree to 5 = strongly agree Range: 21–105 (Sum of items)	Higher score indicates better safety climate
Perceived Conflict of Interest	Conflict of Interest Scale	6 items, 1 = strongly disagree to 4 = strongly agree Range: 6–24 (Sum of items)	Higher score indicates higher conflict of interest
Interpersonal Influence	Interpersonal Norms	4 items, importance to others of using PPE, 0 = not at all, 1 = sort of, 2 = a lot Range: 0–2 (Mean)	Higher score indicates higher belief that others think PPE is important
	Interpersonal Modeling	3 items, frequency of others’ use of PPE, 0 = never to 3 = usually Range: 0–3 (Mean)	Higher score indicates higher use of PPE by co-workers
	Total interpersonal influence = mean of seven items (range 0–3)		

Legends: PPE = Personal Protective Equipment, HDHQ = Hazardous Drug Handling Questionnaire

### Statistical analysis

The Statistical Package for the Social Sciences version 28 was used for data analysis. Returned questionnaires were examined for completeness, with no missing data detected. Continuous variables were summarized using means (M) and standard deviations (SD), whereas frequencies and percentages represented categorical variables. Kolmogorov–Smirnov normality testing indicated an approximately normal distribution; therefore, parametric analyses were performed. Independent samples t-tests were conducted to compare the means of two groups, while one-way Analysis of Variance (ANOVA) evaluated the differences among three or more groups.

To examine the relationships between independent variables and PPE compliance, Pearson bivariate correlations were conducted to assess both the strength and direction of associations between each pair of variables. Because the study aimed to explore pairwise relationships rather than to develop a predictive model, multivariable regression analyses were not performed. Nevertheless, the proportion of variance explained by each bivariate correlation can be approximated by squaring the correlation coefficient (r^2^), providing insight into each independent variable’s relative contribution. A *p*-value of ≤ 0.05 was considered statistically significant.

## Results

### Participant characteristics

One hundred nurses completed the questionnaire (*n* = 100; response rate 68.5%). Among respondents, 51% were male and 49% female. Forty-five percent were aged 33–43 years. Over half (55%) worked in non-profit hospitals, and 56% were employed in inpatient departments. Although 54% were not oncology-certified nurses, 43% reported 6–10 years of oncology experience. Most respondents (79%) had received formal PPE training in the workplace. Participant characteristics are summarized in Table 2.

**Table 2:** Participant characteristics

Characteristics	N (%)
Age	
22–32 years	42 (42%)
33–43 years	45 (45%)
44–54 years	10 (10%)
55–65 years	3 (3%)
Gender	
Male	51 (51%)
Female	49 (49%)
Educational Level	
Bachelor’s degree	92 (92%)
Master’s degree	6 (6%)
Other	2 (2%)
Place of Work	
Non-profit Hospitals	55 (55%)
Private Hospitals	12 (12%)
Public Hospitals	33 (33%)
Job Title	
Head Nurse & Assistant Head Nurse	16 (16%)
Registered Nurse	84 (84%)
Work Shift	
Morning	31 (31%)
Evening	12 (12%)
Night	2 (2%)
Rotation	55 (55%)
Type of Setting	
Inpatient	56 (56%)
Outpatient	41 (41%)
Both	3 (3%)
Nursing Experience	
1–5 years	23 (23%)
6–10 years	35 (35%)
11–15 years	22 (22%)
16–20 years	10 (10%)
> 20 Years	10 (10%)
Oncology Nursing Experience	
1–5 Years	35 (35%)
6–10 Years	43 (43%)
11–15 Years	13 (13%)
16–20 Years	9 (9%)
Chemotherapy Handling Experience	
1–5 Years	38 (38%)
6–10 Years	41 (41%)
11–15 Years	13 (13%)
16–20 Years	8 (8%)
Number of patients for whom the nurse administers chemotherapy per day	
1–5	64 (64%)
6–10	26 (26%)
11–15	8 (8%)
16–20	2 (2%)
Daily number of patients receiving chemotherapy at workplace	
1–5	12 (12%)
6–10	34 (34%)
11–15	21 (21%)
16–20	13 (13%)
> 20	20 (20%)
Oncology Certification/Specialization Course	
Certified	46 (46%)
Not certified	54 (54%)
Formal On-the-Job PPE Training	
Yes	79 (79%)
No	21 (21%)

### Factors influencing compliance with PPE

Knowledge regarding chemotherapy exposure was insufficient (*M* = 6.77, SD = 1.57) with an observed range of 2–11. According to Dr. Polovich, a score below 7 correct responses out of 12 indicates inadequate knowledge.

Some misconceptions were common among participants. For example, 25% incorrectly believed the statement, “Chemotherapy cannot enter the body through contact with contaminated surfaces,” to be true. Additionally, 27 and 33% of participants incorrectly identified the following statements as true: “A surgical mask provides protection from chemotherapy aerosols,” and “Alcohol hand sanitizer is as effective as soap and water in removing chemotherapy residue,” respectively.

Perceived barrier to PPE use were moderate (*M* = 29.93, SD = 9.00). The most frequently reported barriers were: “PPE is too expensive to use it all the time” (*M* = 3.12, SD = 0.96), “PPE makes me feel too hot” (*M* = 3.09, SD = 0.86), “Others around me don’t use PPE” (*M* = 2.42, SD = 0.99), and “I don’t have the time to use PPE” (*M* = 2.33, SD = 0.85).

Regarding interpersonal influences, nurses scored high on the Interpersonal Modeling subscale (*M* = 2.64, SD = 0.41) and moderate on the Interpersonal Norms subscale (*M* = 0.70, SD = 0.67). The overall interpersonal influence score, calculated as the mean of all items across both subscales, was moderate (*M* = 1.53, SD = 0.57).

Self-efficacy for PPE use was low (*M* = 13.22, SD = 3.32), with the lowest-ranked item being “I am confident that I can use PPE properly” (*M* = 1.41, SD = 0.53).

Perceived risk of harm from chemotherapy exposure was moderate (*M* = 2.17, SD = 0.54), with the lowest-scoring item: “Exposure to chemotherapy is a serious problem at work” (*M* = 1.42, SD = 0.57).

Perceived conflict of interest between self-protection and patient care was low (*M* = 10.90, SD = 3.37), with the lowest-scoring item: “PPE keeps me from doing my job to the best of my abilities” (*M* = 1.59, SD = 0.69).

The perceived workplace safety climate was moderate (*M* = 54.11, SD = 11.43). The lowest-scoring items were: “In my work area we are expected to comply with safe-handling policies and procedures” (*M* = 2.26, SD = 0.82) and “Employees are encouraged to become involved in safety and health matters” (*M* = 2.29, SD = 0.72).

The vast majority of nurses (95%) reported that written chemotherapy policies existed in their workplace. For detailed results, please refer to Additional File 4.

Compliance with PPE was greatest during drug administration (*M* = 2.41, SD = 0.68) and lowest during disposal (*M* = 2.2, SD = 0.90). Overall compliance across all exposure activities (administration, disposal, and handling excreta) was moderate (*M* = 2.3, SD = 0.67) (Table 3).

**Table 3:** Compliance with PPE during various drug activities

Use of Chemotherapy Handling Precautions	Administration *N* = 100 M (SD)	Disposal *N* = 96 M (SD)	Handling Excreta *N* = 87 M (SD)
Closed System Transfer Devices	2.9 (1.2)	-	-
Gloves labeled for use with chemotherapy	2.7 (1.3)	2.7 (1.6)	2.2 (1.5)
Other gloves (e.g., vinyl)	2.1 (1.1)	2.3 (1.2)	2.3 (1.4)
Double gloves	2.7 (1.3)	2.4 (1.6)	2.5 (1.4)
Gowns labeled for use with chemotherapy	3.4 (1.2)	2.8 (1.5)	2.4 (1.5)
Other gowns (e.g., isolation, lab coats)	2.2 (1.4)	2.2 (1.4)	2.3 (1.6)
Do you re-use disposable gowns?	1.9 (1.6)	1.7 (1.7)	1.7 (1.8)
Eye protection	1.5 (1.8)	1.7 (1.9)	2.1 (2.1)
Respirator/mask	2.0 (1.9)	1.9 (1.8)	2.1 (2.0)
Sum	2.41 (0.68)	2.2 (0.90)	2.2 (0.96)
Total level of compliance during all three activities	M = 2.3, SD = 0.67		

Legends:Scoring: Low = (0.0 -1.66), Moderate = (1.67–3.33), High = (3.34–5.0). *M* = mean; SD = standard deviation

### Association between compliance with PPE and general characteristics

There were no significant associations between compliance and demographic characteristics (Table 4), except for nurses certified in oncology (*p* = 0.005) and those who had received on-the-job PPE training (*p* = 0.03).

**Table 4:** Association between the compliance and demographics

Characteristic	M (SD)	*t/F*-values	*p* value	Test Used
Age				
22–32 years	2.26 (0.62)	0.80	0.49	One-way ANOVA
33–43 years	2.31 (0.72)			
44–54 years	2.50 (0.59)			
55–65 years	1.84 (1.84)			
Gender				
Male	2.33 (0.68)	0.49	0.62	Independent T-Test
Female	2.26 (0.67)			
Educational Level				
Bachelor’s degree	2.29 (0.68)	0.57	0.56	One-way ANOVA
Master’s degree	2.14 (0.62)			
Other	2.73 (0.73)			
Place of Work				
Non-Profit Hospitals	2.31 (0.93)	0.65	0.52	One-way ANOVA
Private Hospitals	2.23 (0.62)			
Public Hospitals	2.40 (0.64)			
Job Title				
Head Nurse & Assistant HN	2.34 (0.68)	1.50	0.13	Independent T-Test
Registered Nurse	2.06 (0.53)			
Work Shift				
Morning	2.05 (0.53)	1.98	0.12	One-way ANOVA
Evening	2.46 (0.50)			
Night	2.45 (0.61)			
Rotations	2.40 (0.74)			
Type of Setting				
Inpatient	2.24 (0.64)	1.36	0.26	One-way ANOVA
Outpatient	2.40 (0.71)			
Both	1.82 (0.14)			
Nursing Experience				
1–5 years	2.33 (0.63)	0.83	0.50	One-way ANOVA
6–10 years	2.14 (0.60)			
11–15 years	2.45 (0.77)			
16–20 years	2.29 (0.76)			
> 20 Years	2.41 (0.66)			
Oncology Nursing Experience				
1–5 Years	2.26 (0.57)	0.60	0.61	One-way ANOVA
6–10 Years	2.32 (0.73)			
11–15 Years	2.13 (0.74)			
16–20 Years	2.51 (0.61)			
Chemotherapy Handling Experience				
1–5 Years	2.34 (0.65)	0.62	0.60	One-way ANOVA
6–10 Years	2.31 (0.71)			
11–15 Years	2.06 (0.64)			
16–20 Years	2.40 (0.55)			
Number of patients for whom the nurse administers chemotherapy per day				
1–5	2.31 (0.64)	0.31	0.81	One-way ANOVA
6–10	2.32 (0.79)			
11–15	2.10 (0.60)			
16–20	2.11 (0.15)			
Number of patients receiving chemotherapy per day				
1–5	2.15 (0.58)	0.55	0.69	One-way ANOVA
6–10	2.34 (0.71)			
11–15	2.40 (0.69)			
16–20	2.37 (0.77)			
> 20	2.15 (0.56)			
Certification in Oncology Nursing				
Certified	2.47 (0.69)	2.89	0.005	Independent T Test
Not certified	2.09 (0.58)			
Training on PPE use				
Yes	2.57 (0.56)	2.12	0.03	Independent T Test
No	2.22 (0.68)			

Legends: *M* = mean; SD = standard deviation; *p* = probability value; *t* = t statistic comparing group means; *F* = F statistic comparing variance between groups; ANOVA = analysis of variance; HN = head nurse

### Correlation between compliance with PPE and Independent variables

The correlations between independent variables and PPE compliance varied in strength and direction. The proportion of variance explained by each significant bivariate correlation was estimated using r^2^. For example, self-efficacy showed a strong negative correlation with perceived risk (*r* = −0.67, *p* = 0.006, r^2^ = 0.45), indicating that participants with higher self-efficacy perceived lower risk, with 45% of the variance in perceived risk explained by self-efficacy. Level of knowledge moderately negatively correlated with perceived barriers (*r* = −0.49, *p* = 0.003, r^2^ = 0.24), suggesting that more knowledgeable participants perceived fewer barriers, accounting for 24% of the variance. Level of Knowledge also showed a moderate positive correlation with interpersonal influences (*r* = 0.58, *p* = 0.04, r^2^ = 0.34), indicating that participants with higher level of knowledge were more influenced by peers and supervisors, explaining 34% of the variance. Finally, Workplace Safety Climate was moderately positively correlated with Self-Efficacy (*r* = 0.53, *p* < 0.001, r^2^ = 0.28), suggesting that more confident participants perceived a safer work environment, accounting for 28% of the variance (Table 5).

**Table 5:** Correlation between compliance and Independent variables

Items	Level of Knowledge	Self-Efficacy	Perceived Barriers	Perceived Risk	Interpersonal Influences	Conflict of Interest	Workplace Safety Climate
Self-Efficacy	−0.016 (0.10)	-	-	-	-	-	-
Perceived Barriers	−0.49 (0.003*)	−0.37 (0.001*)	-	-	-	-	-
Perceived Risk	0.04 (0.62)	−0.67 (0.006*)	0.39 (0.00*)	-	-	-	-
Interpersonal Influences	0.58 (0.04*)	0.22 (0.023*)	−0.21 (0.38)	0.09 (0.343)	-	-	-
Conflict of Interest	−0.288 (0.004*)	−0.06 (0.540)	0.27 (0.006*)	−0.07 (0.444)	−0.35 (0.000*)	-	-
Workplace Safety Climate	0.11 (0.257)	0.53 (0.000*)	−0.36 (0.000*)	−0.24 (0.014*)	0.13 (0.171)	−0.12 (0.233)	-
Total Level of Compliance Score	0.17 (0.084)	0.16 (0.100)	−0.39 (0.000*)	0.01 (0.919)	0.31 (0.001*)	−0.15 (0.137)	0.11 (0.259)

Legends:Positive values signify a direct correlation, whereas negative values signify an inverse relationship

## Discussion

This study assessed PPE compliance among oncology nurses in Palestine and identified associated influencing factors.

Overall, nurses demonstrated moderate compliance when handling chemotherapy, with the highest compliance during administration and the lowest during disposal. This moderate level suggests that oncology nurses are not fully maximizing the available safety measures and, therefore, remain at risk. These findings are consistent with those of Chen et al. [18], who reported lower compliance during disposal and handling of excreta. Nurses may perceive these tasks as involving lower drug concentrations and, therefore, lower exposure risk. However, chemotherapy is hazardous during all handling activities, and strict compliance should be maintained.

Nurses in this study demonstrated insufficient knowledge regarding chemotherapy exposure risks, indicating a need for ongoing education and refresher training to ensure adherence to current best practices. Hospital administrators should support nurses in pursuing specialization or advanced training, enabling them to share expertise with colleagues. In Palestine, not all institutions provide continuing education, and oncology nurses often gain expertise once they start working in oncology units. Similar findings were reported by Abu Sharour et al. [22] among Jordanian oncology nurses, linking low knowledge to inadequate education and in-service training.

Moderate perceived barriers were reported, including PPE cost, limited time, lack of role modeling, and discomfort. Callahan et al. [5] also found that protective gowns were perceived as warm and uncomfortable, prompting substitution with isolation gowns or laboratory coats. Lee et al. [23] cited time pressure as a common barrier that creates a conflict between providing patient care and adherence to safety directives. While some barriers, such as the discomfort from PPE, cannot be entirely avoided, managers can involve staff in selecting and trialing different types of PPE and providing feedback on comfort and usability. Additionally, staff, especially those working in private institutions, should not receive direct or indirect communications that discourage PPE use due to cost, because this may constitute negative reinforcement and undermine safety.

Nurses reported low self-efficacy for using PPE, likely related to inadequate knowledge. In line with the theoretical framework [17], knowledge directly influences self-efficacy and perceived barriers. Knowledge also affects perceived exposure risk, which was moderate in this study. If nurses do not perceive risk, they may be less compliant with protective measures, increasing actual exposure.

The workplace safety climate was moderate, reflecting a neutral perception of organizational support. While most participants were aware of written safety standards, health organizations can support better safety climates through policies, adequate supplies, training, and administrative support [5, 7].

Although PPE was readily available (see Additional File 4), some nurses used less-protective alternatives, such as isolation gowns or laboratory coats, suggesting limited awareness of relative protective effectiveness or insufficient supervision. Managers should implement strict monitoring and enforcement of safety procedures.

Total interpersonal influence was moderate, indicating that nurses’ behavior is affected by the actions and expectations of others. Specifically, the high score on the Interpersonal Modeling subscale (see Additional File 4) suggests that nurses are more likely to use PPE when coworkers demonstrate correct practices. In contrast, Interpersonal Norms were moderate, indicating that perceived social expectations had a lesser impact than direct modeling. This agrees with findings by Polovich and Clark [24] and Callahan et al. [5], who reported higher PPE use when coworkers consistently adhered to recommended precautions.

A low perceived conflict between PPE use and patient care was reported, suggesting that nurses were able to deliver care without compromising their safety, likely due to adequate staffing levels.

No significant associations were found between compliance and demographic characteristics, except for oncology certification and receipt of on-the-job training. In contrast, Chen et al. [18] found higher compliance among more experienced nurses and those working in cancer departments. Furthermore, Abu Sharour et al. [22] reported higher compliance among younger nurses, who may be more eager to apply best practices.

Although the study was conducted in the West Bank of Palestine, the findings may be transferable to other low- and middle-income countries with similar healthcare systems and resource constraints.

### Limitations

The study had several limitations. First, it focused exclusively on registered nurses who handle chemotherapy, excluding other healthcare and non-healthcare workers who may also be at risk. Second, the study settings were anonymized, limiting comparisons across hospitals. Third, data were not collected from the Gaza Strip, which restricts the generalizability of the findings to all regions of Palestine. Fourth, administering the questionnaire in English among an Arabic-speaking population may have influenced participant responses. Finally, the study used bivariate correlation. Although multivariable models could have provided an understanding of the independent effects of each variable while controlling for confounding factors, such analyses were beyond the scope of this study.

### Recommendations

Nursing programs in Palestine should allocate additional instructional hours to oncology and chemotherapy safety to better prepare graduates for clinical practice. Hospital managers are encouraged to conduct compliance audits, assign experienced nurses to work with less experienced one, and provide individualized performance feedback.

Currently, no registry tracks nurses’ chemotherapy exposure or links exposure to negative outcomes, which limits the ability to detect related health effects. Longitudinal and epidemiological studies are recommended to evaluate potential toxicities. Further investigation should incorporate broader and more varied samples to improve generalizability. Additionally, future studies could extend the analysis of this study by using multivariable approaches to examine the combined and independent effects of multiple factors on PPE compliance and related outcomes.

## Conclusion

Occupational exposure to chemotherapy poses a significant risk to healthcare staff, particularly nurses. Proper use of PPE is essential to reduce the risk. This study found that oncology nurses in Palestine demonstrated moderate PPE compliance, indicating ongoing vulnerability to toxic exposure. Key influencing factors included insufficient knowledge, perceived barriers, low self-efficacy for using PPE, moderate perceived risk, and a moderate workplace safety climate. Improving compliance requires continuing education, participation in available training programs, and developing a supportive workplace culture through routine monitoring and supervision. The findings provide a foundation for future research and can guide policymakers and healthcare leaders in strengthening chemotherapy safety standards and monitoring compliance across Palestinian healthcare settings.

## Electronic supplementary material

Below is the link to the electronic supplementary material.

Supplementary Material 1

## Data Availability

Data and materials are provided within the manuscript and supplementary information files.
