# Supplementary material for: Nurse compliance with personal protective equipment when handling chemotherapy: a multicenter cross-sectional study in Palestine
Source: BMC Health Serv Res. 2026 Feb 3;26:235. doi: 10.1186/s12913-026-14132-x (PMC12903596; doi:10.1186/s12913-026-14132-x)
Supplement: Supplementary file 1 — Supplementary Material 1 [file 12913_2026_14132_MOESM1_ESM.docx]

**Additional File 1**: The Hazardous Drugs Handling Questionnaire

Thank you for agreeing to participate in this study of nurses who handle chemotherapy. “Handling” refers to chemotherapy preparation, administration, disposal, and coming into contact with patient’s excreta that may be contaminated with chemotherapy.

- By preparation, we mean transferring chemotherapy drugs from vials or ampoules to syringes or IV containers.
- By administration, we mean giving chemotherapy to patients by IV, injection, orally, etc.
- By disposal, we mean discarding equipment used in chemotherapy preparation or administration.
- By handling excreta, we mean emptying bedpans, urinals or emesis basins.

Do you personally handle chemotherapy at work, either chemotherapy preparation or administration?

 Yes

 No → If you answered “No” STOP HERE and return the questionnaire.

If you answered “Yes”:

1. Please read each item carefully.
2. Place a check in the box next to your selection from the list of options.
3. Please answer all of the questions that apply to your chemotherapy handling.

**Section A**: Sociodemographic Information

| 1. Age in years: ___________________ | | | | | | |
| --- | --- | --- | --- | --- | --- | --- |
| 1. Gender | a.  Male | | | | b.  Female | |
| 1. Highest level of Nursing education | a.  Diploma  b.  Bachelor’s degree | | | c.  Master’s degree  d.  Other ___________ | | |
| 1. Place of work | a.  Augusta Victoria  b.  Istishari Hospital  c.  Beit Jala Hospital | | | d.  Al – Watani Hospital  e.  Al-Najah Hospital  f.  Palestine Hospital (Harmala) | | |
| 1. Job title | a.  Head Nurse  b.  Assistant Head Nurse | | | | c.  Registered Nurse  d.  Licensed Practical  Nurse | |
| 1. Work shift | a.  Morning | | b.  Evening | | | c.  Night |
| 1. In what type of setting you do handle chemotherapy? | a.  Adult Inpatient  b.  Pediatric Inpatient | | | c.  Outpatient  d.  Day care | | |
| 1. Years of Nursing experience _______________ | | | | | | |
| 1. Years of Oncology Nursing experience _____________ | | | | | | |
| 1. Years of Chemotherapy Handling experience ____________ | | | | | | |
| 1. Number of patients for whom you personally  prepare and/or administer chemotherapy per day _____________ | | | | | | |
| 1. Number of patients receiving chemotherapy  per day at your work place: _______________ | | | | | | |
| 1. Are you certified in chemotherapy administration by a professional organization? | | a.  Certified  b.  Not certified  If certified, state the name of the organization _____________________________ | | | | |
| 1. Have you received formal training on how to use PPE? | | a.  Yes  b.  No | | | | |

Section B: Chemotherapy Exposure Knowledge

| Select one answer to each of the following statements about chemotherapy exposure: | True | False | Don’t Know |
| --- | --- | --- | --- |
| 1. Chemotherapy can enter the body through breathing it in |  |  |  |
| 1. Chemotherapy can enter the body through ingesting it |  |  |  |
| 1. Chemotherapy can enter the body through contact with contaminated surfaces |  |  |  |
| 1. Chemotherapy can enter the body through contact with spills and splashes |  |  |  |
| 1. Chemotherapy gas and vapor in air can enter the body through skin and mucous membranes |  |  |  |
| 1. Oral forms of chemotherapy do not have the potential to be absorbed |  |  |  |
| 1. Chemotherapy in liquid form can be absorbed through the skin |  |  |  |
| 1. A surgical mask provides protection from chemotherapy aerosols |  |  |  |
| 1. All types of gloves provide the same level of protection |  |  |  |
| 1. Chemotherapy can more easily enter the body through damaged skin |  |  |  |
| 1. Alcohol hand sanitizer is as effective as soap and water in removing chemotherapy residue |  |  |  |
| 1. Chemotherapy can enter the body through contaminated foods, beverages, or cosmetics |  |  |  |

Section C: Self-efficacy for Using PPE
Indicate your level of agreement with each of these statements about using personal protective equipment (PPE) when handling chemotherapy (SA = Strongly Agree; A = Agree; D = Disagree; SD = Strongly Disagree):

|  | SA | A | D | SD |
| --- | --- | --- | --- | --- |
| 1. I am confident that I can use PPE properly |  |  |  |  |
| 1. I am confident that I can protect myself from chemotherapy exposure |  |  |  |  |
| 1. I am given enough information on how to protect myself from chemotherapy exposure |  |  |  |  |
| 1. My supervisor goes out of his/her way to make sure I am protected |  |  |  |  |
| 1. Reuse of disposable PPE makes me feel less protected |  |  |  |  |
| 1. I am provided with the best available PPE |  |  |  |  |
| 1. My supervisor goes out of his/her way to make sure I am provided with proper fitting PPE |  |  |  |  |

Section D:

D.1. Does your workplace have written policies and/or procedures for handling chemotherapy?

- 1.  Yes
  2.  No

| D.2. Where is chemotherapy prepared in your workplace? | |
| --- | --- |
| 1. Pharmacy |  |
| 1. Specially designated room separate from the patient care area |  |
| 1. Area within the patient treatment area / room |  |
| 1. Other (specify) ________________________ |  |

| D.3. What personal protective equipment is available for performing the following chemotherapy handling activities? Check all that apply. | | | | |
| --- | --- | --- | --- | --- |
| Activity | Gloves | Gowns | Eye  Protection | Respirator/ Mask |
| 1. Administration |  |  |  |  |
| 1. Handling Excreta |  |  |  |  |
| 1. Disposal |  |  |  |  |
| 1. Cleaning Spills |  |  |  |  |

Section E: Chemotherapy Administration:

E.1. Are you responsible for administering chemotherapy?

 Yes  No → If you answered “No” proceed to Section F.

Complete this section ONLY if you administer chemotherapy.
 E.2. What type of gloves do you wear while administering chemotherapy?

| - 1. None |  |
| --- | --- |
| - 1. Chemotherapy-designated gloves |  |
| - 1. Vinyl (polyvinyl chloride, PVC) |  |
| - 1. Latex examination gloves |  |
| - 1. Sterile surgical gloves |  |
| - 1. Other (specify) ____________________ |  |

E.3. What type of protective clothing do you wear while administering chemotherapy?

Check all that apply.

| - 1. None |  |
| --- | --- |
| - 1. Chemotherapy-designated gown |  |
| - 1. Personal lab coat |  |
| - 1. Lab coat provided by office |  |
| - 1. Cloth gown |  |
| - 1. Isolation gown |  |
| - 1. Other (specify) _____________________ |  |

E.4. Please indicate how much of the time you use the following while administering

chemotherapy:

| \|  \| Always \| 76-99% \| 51-75% \| 26-50% \| 1-25% \| Never \| \| --- \| --- \| --- \| --- \| --- \| --- \| --- \| \| Closed system transfer device \|  \|  \|  \|  \|  \|  \| \| Gloves labeled for use with chemotherapy \|  \|  \|  \|  \|  \|  \| \| Other gloves (e.g. vinyl) \|  \|  \|  \|  \|  \|  \| \| Double gloves \|  \|  \|  \|  \|  \|  \| \| Gowns labeled for use with chemotherapy \|  \|  \|  \|  \|  \|  \| \| Other gowns (e.g. isolation) \|  \|  \|  \|  \|  \|  \| \| Do you re-use disposable gowns? \|  \|  \|  \|  \|  \|  \| \| Eye protection \|  \|  \|  \|  \|  \|  \| \| Respirator/mask \|  \|  \|  \|  \|  \|  \| |
| --- | --- | --- | --- | --- | --- | --- | --- | --- | --- | --- | --- | --- | --- | --- | --- | --- | --- | --- | --- | --- | --- | --- | --- | --- | --- | --- | --- | --- | --- | --- | --- | --- | --- | --- | --- | --- | --- | --- | --- | --- | --- | --- | --- | --- | --- | --- | --- | --- | --- | --- | --- | --- | --- | --- | --- | --- | --- | --- | --- | --- | --- | --- | --- | --- | --- | --- | --- | --- | --- | --- |

Section F: Chemotherapy Disposal:

F.1. Are you responsible for disposing of chemotherapy?

 Yes  No → If you answered “No” proceed to Section G.

Complete this section ONLY if you dispose of chemotherapy.
 F.2. Please indicate how much of the time you use the following when disposing

of chemotherapy:

| \|  \| Always \| 76-99% \| 51-75% \| 26-50% \| 1-25% \| Never \| \| --- \| --- \| --- \| --- \| --- \| --- \| --- \| \| 1. Gloves labeled for use with chemotherapy \|  \|  \|  \|  \|  \|  \| \| 1. Other gloves (e.g. vinyl) \|  \|  \|  \|  \|  \|  \| \| 1. Double gloves \|  \|  \|  \|  \|  \|  \| \| 1. Gowns labeled for use with chemotherapy \|  \|  \|  \|  \|  \|  \| \| 1. Other gowns (e.g. isolation) \|  \|  \|  \|  \|  \|  \| \| 1. Do you re-use disposable gowns? \|  \|  \|  \|  \|  \|  \| \| 1. Eye protection \|  \|  \|  \|  \|  \|  \| \| 1. Respirator/mask \|  \|  \|  \|  \|  \|  \| |
| --- | --- | --- | --- | --- | --- | --- | --- | --- | --- | --- | --- | --- | --- | --- | --- | --- | --- | --- | --- | --- | --- | --- | --- | --- | --- | --- | --- | --- | --- | --- | --- | --- | --- | --- | --- | --- | --- | --- | --- | --- | --- | --- | --- | --- | --- | --- | --- | --- | --- | --- | --- | --- | --- | --- | --- | --- | --- | --- | --- | --- | --- | --- | --- |

Section G: Handling Contaminated Excreta:

G.1. Are you responsible for handling chemotherapy-contaminated excreta?

 Yes  No → If you answered “No” proceed to Section H.

| Complete this section ONLY if you handle chemotherapy-contaminated excreta.  G.2. Please indicate how much of the time you use the following when handling excreta:   \|  \| Always \| 76-99% \| 51-75% \| 26-50% \| 1-25% \| Never \| \| --- \| --- \| --- \| --- \| --- \| --- \| --- \| \| 1. Gloves labeled for use with chemotherapy \|  \|  \|  \|  \|  \|  \| \| 1. Other gloves (e.g. vinyl) \|  \|  \|  \|  \|  \|  \| \| 1. Double gloves \|  \|  \|  \|  \|  \|  \| \| 1. Gowns labeled for use with chemotherapy \|  \|  \|  \|  \|  \|  \| \| 1. Other gowns (e.g. isolation) \|  \|  \|  \|  \|  \|  \| \| 1. Do you re-use disposable gowns? \|  \|  \|  \|  \|  \|  \| \| 1. Eye protection \|  \|  \|  \|  \|  \|  \| \| 1. Respirator/mask \|  \|  \|  \|  \|  \|  \| |
| --- | --- | --- | --- | --- | --- | --- | --- | --- | --- | --- | --- | --- | --- | --- | --- | --- | --- | --- | --- | --- | --- | --- | --- | --- | --- | --- | --- | --- | --- | --- | --- | --- | --- | --- | --- | --- | --- | --- | --- | --- | --- | --- | --- | --- | --- | --- | --- | --- | --- | --- | --- | --- | --- | --- | --- | --- | --- | --- | --- | --- | --- | --- | --- |

Section H:

| H.1. Are chemotherapy spill kits available in your work area? |  Yes |  No | |
| --- | --- | --- | --- |
| H.2. During the most recent chemotherapy spill in your workplace, did you use the materials in the spill kit? |  Yes |  No |  N/A |

H.3. Please write the name of three chemotherapy drugs that you handle most frequently:

1.________________________2. ___________________________3. _____________________

Section I: Barriers to Using PPE

I.1. Indicate your level of agreement with each of the following statements (SA = Strongly Agree; A = Agree; D = Disagree; SD = Strongly Disagree):

| Some reasons that I may not wear PPE regularly when handling chemotherapy are: | SA | A | D | SD |
| --- | --- | --- | --- | --- |
| 1. I don’t think PPE is necessary |  |  |  |  |
| 1. I don’t think PPE works |  |  |  |  |
| 1. I don’t have the time to use PPE |  |  |  |  |
| 1. I was not trained to use PPE. |  |  |  |  |
| 1. PPE is uncomfortable to wear |  |  |  |  |
| 1. PPE makes it harder to get the job done |  |  |  |  |
| 1. PPE is not always available |  |  |  |  |
| 1. Others around me don’t use PPE |  |  |  |  |
| 1. There is no policy requiring PPE |  |  |  |  |
| 1. People would think I am overly cautious |  |  |  |  |
| 1. It is hard to get chemotherapy-designated PPE |  |  |  |  |
| 1. PPE is too expensive to use it all the time |  |  |  |  |
| 1. PPE makes me feel too hot |  |  |  |  |

Section J: Risks of Chemotherapy Exposure

J.1. Indicate your level of agreement with each of the following statements about the risks of chemotherapy exposure (SA = Strongly Agree; A = Agree; D = Disagree; SD = Strongly Disagree)

|  | SA | A | D | SD |
| --- | --- | --- | --- | --- |
| 1. Exposure to chemotherapy is a serious problem at work |  |  |  |  |
| 1. I am concerned about chemotherapy exposure at work and how it might affect my health |  |  |  |  |
| 1. Compared to co-workers, my chance of harm from chemotherapy exposure is lower |  |  |  |  |
| 1. If exposed to chemotherapy, there is a real chance that I might experience side effects |  |  |  |  |
| 1. Chemotherapy exposure is not as harmful as some people claim. |  |  |  |  |
| 1. Compared to other work-related health risks, chemotherapy exposure is less serious |  |  |  |  |
| 1. I am not worried about future negative health effects from chemotherapy exposure |  |  |  |  |

Section K Interpersonal Influence

Part 1: Interpersonal Norms

K.1. How often do the following people wear personal protective equipment when handling chemotherapy?

|  | Never | Sometimes | About  Half | Usually | Does not apply |
| --- | --- | --- | --- | --- | --- |
| 1. Your co-workers |  |  |  |  |  |
| 1. Other nurses you know |  |  |  |  |  |
| 1. Oncology nurses in general |  |  |  |  |  |

Part B: Interpersonal Modeling

K.2. According to the following people, how important is wearing PPE when handling chemotherapy?

|  | Not at all important | Sort Of important | Very important | Does not  apply |
| --- | --- | --- | --- | --- |
| 1. Your co-workers |  |  |  |  |
| 1. Other nurses you know |  |  |  |  |
| 1. Your supervisor or manager |  |  |  |  |
| 1. Your employer |  |  |  |  |

Section L: Conflict of Interest Scale

Indicate your level of agreement with each of the following statements:

SA = Strongly Agree; A = Agree; D = Disagree; SD = Strongly Disagree:

|  | SA | A | D | SD |
| --- | --- | --- | --- | --- |
| 1. Personal protective equipment keeps me from doing my job to the best of my abilities. |  |  |  |  |
| 1. Wearing personal protective equipment makes my patients worry. |  |  |  |  |
| 1. Patient care often interferes with my being able to comply with using precautions. |  |  |  |  |
| 1. I cannot always use safe handling precautions because patient’s needs come first. |  |  |  |  |
| 1. Sometimes I have to choose between wearing PPE and caring for my patients. |  |  |  |  |
| 1. Wearing personal protective equipment makes my patients feel uncomfortable. |  |  |  |  |

Section M: Workplace Safety Climate

M1. Indicate your level of agreement with these statements regarding safety in your work place (SA = Strongly Agree; A = Agree; N = Neutral; D = Disagree; SD = Strongly Disagree):

|  | SA | A | N | D | SD |
| --- | --- | --- | --- | --- | --- |
| 1. Chemotherapy gloves are readily accessible in my work area |  |  |  |  |  |
| 1. Chemotherapy gowns are readily available in my work area |  |  |  |  |  |
| 1. The protection of workers from occupational exposure to chemotherapy is a high priority with management where I work |  |  |  |  |  |
| 1. On my unit, all reasonable steps are taken to minimize hazardous job tasks |  |  |  |  |  |
| 1. Employees are encouraged to become involved in safety and health matters |  |  |  |  |  |
| 1. Managers on my unit make sure employees are protected from occupational exposure to chemotherapy |  |  |  |  |  |
| 1. My job duties do not often interfere with my being able to follow chemotherapy safe handling precautions |  |  |  |  |  |
| 1. I have enough time in my work to always follow chemotherapy safe handling precautions |  |  |  |  |  |
| 1. I usually do not have too much to do so that I can follow chemotherapy safe handling precautions |  |  |  |  |  |
| 1. On my unit, unsafe work practices are corrected by supervisors |  |  |  |  |  |
| 1. My supervisor talks to me about safe work practices |  |  |  |  |  |
| 1. I have had the opportunity to be properly trained to use personal protective equipment so that I can protect myself from chemotherapy exposures |  |  |  |  |  |
| 1. Employees are taught to be aware of and to recognize potential health hazards at work |  |  |  |  |  |
| 1. In my work area, I have access to policies and procedures regarding safety |  |  |  |  |  |
| 1. My work area is kept clean |  |  |  |  |  |
| 1. My work area is not cluttered |  |  |  |  |  |
| 1. My work area is not crowded |  |  |  |  |  |
| 1. There is minimal conflict within my work area |  |  |  |  |  |
| 1. The members of my work area support each another |  |  |  |  |  |
| 1. In my work area, there is open communication between supervisors and staff |  |  |  |  |  |
| 1. In my work area we are expected to comply with safe handling policies and procedures |  |  |  |  |  |

Thank you
